# The Rise in Divorce and Cohabitation: Is There a Link?

**DOI:** 10.1111/padr.12063

**Published:** 2017-06-05

**Authors:** Brienna Perelli‐Harris, Ann Berrington, Nora Sánchez Gassen, Paulina Galezewska, Jennifer A. Holland

increases in divorce and cohabitation are among the most prominent behavioral changes to affect the family over the past few decades. Both behaviors have received substantial attention, with some commentators referring to them as indicative of a breakdown in the family. Undoubtedly, the two behaviors have fundamentally altered the institution of marriage. The increase in divorce has changed marriage from a union intended to be life‐long to a relationship that has the potential to dissolve. At the same time, cohabitation has emerged as a way for two people to live together without marriage and to avoid the potentially higher costs of divorce if the union does not last. Thus, divorce and cohabitation appear to be intrinsically linked.

Current theories explaining the emergence of these behaviors tend either to explain divorce and cohabitation separately or to include them in a broader set of changing family behaviors, often referred to as the second demographic transition (Sobotka 2008; Lesthaeghe 2010; van de Kaa 2001). These theories usually point to economic shifts (Becker 1991; Oppenheimer 1997; Ruggles 2015) or social and ideational change (Giddens 1992; Lesthaeghe 2010; Perelli‐Harris et al. 2010) to explain their emergence. While these theories are crucial for understanding the underlying factors leading to the behaviors, they have not specifically examined whether or how the increase in divorce may have been fundamental to the development of cohabitation. Given the dramatic increase in both divorce and cohabitation throughout much of the industrialized world, we argue that the rise in divorce could be an important catalyst for the increase in cohabitation.

The relationship between divorce and cohabitation is most likely not unidirectional. Instead, they could influence each other through feedback loops (Bumpass 1990). For example, the experience of cohabitation as a less permanent relationship may lead to greater union instability in general (Berrington and Diamond 1999; Liefbroer and Dourleijn 2006). Furthermore, the emergence of cohabitation may have led to greater selection into marriage, resulting in a decline or stabilization of divorce, as appears to have occurred in the US (Kennedy and Ruggles 2014) and the UK (Beaujouan and Ní Bhrolcháin 2014). While the development of these trends may be complicated, an investigation into whether or how the increase in divorce may have led to the rise of cohabitation is warranted.

To evaluate the evidence in support of a link between divorce and cohabitation, we search for trends and mechanisms at different hierarchical levels: the macro, meso, and micro. Demographers often study processes that occur at the population level (the macro) while also recognizing the importance of decisions made at the individual level (the micro) (Billari 2015). An intermediate level between the two is the family (the meso), often referred to as the intergenerational transmission of behaviors and attitudes. Studying evidence at each of these levels may produce a different understanding of how the link between divorce and cohabitation operates and of the mechanisms through which the two are linked.

To search for evidence and mechanisms, we first use qualitative methods to describe social discourses related to cohabitation and marriage, which can elucidate potential mechanisms and provide explanations for the link. The qualitative evidence comes from focus group data collected in eight European countries and emerged from a broader project that studied the meaning of cohabitation and marriage (see *Demographic Research 2015–16*, Special Collection 17: Focus on Partnerships). The issue of divorce arose in nearly every focus group, especially with respect to how cohabitation is useful as a testing ground to avoid divorce (Perelli‐Harris et al. 2014).

We then analyze quantitative data to see whether evidence supports the idea that the increase in divorce fueled the increase in cohabitation. We draw on official sources and harmonized partnership histories based on surveys in 16 European countries. Since information on cohabitation was not collected in population registers before the 2000s, nationally representative surveys are the only source of detailed cohabitation histories dating back several decades. We also include information on legal reform, as changes in the legal availability of divorce helped to facilitate its increase. Although our analyses cannot conclusively demonstrate causality, they provide insights into whether the evidence is consistent with a direct link between divorce and cohabitation. Given that cohabitation has different meanings in different countries (Perelli‐Harris et al. 2014; Hiekel et al. 2014), we expect the relationship between divorce and cohabitation to be more evident in some countries than in others. In addition, we acknowledge that cohabitation has heterogeneous meanings across individuals, socioeconomic strata, and at different stages of the lifecourse (Perelli‐Harris and Bernardi 2015). While we do not interrogate these meanings here, we believe the divorce revolution has the potential to encourage cohabitation across a range of circumstances—for example, as a precursor or an alternative to marriage (Kiernan 2004; Heuveline and Timberlake 2004).

Finally, we synthesize our findings to suggest how the increase in divorce may have been one of the many factors leading to the increase in cohabitation. Drawing on previous studies and our own results, we elucidate mechanisms at each analytical level—macro, meso, and micro. We argue that divorce may have led to the adoption of cohabitation through the diffusion of new social norms and values about marriage, the process of social learning from parents who divorced, and the personal experience of divorce.

## Data

### Qualitative data

The focus group project sought a better understanding of the increase in cohabitation throughout Europe. The goal of focus group research is not to provide representative data, but to understand general concepts and substantive explanations for social phenomena. Collaborators conducted 7–8 focus groups in the following cities: Vienna, Austria (Berghammer et al. 2014), Florence, Italy (Vignoli and Salvini 2014), Rotterdam, the Netherlands (Hiekel and Keizer 2015), Oslo, Norway (Lappegård and Noack 2015), Warsaw, Poland (Mynarska et al. 2014), Moscow, Russia (Isupova 2015), Southampton, United Kingdom (Berrington et al. 2015), and Rostock and Lubeck, Germany (Klärner 2015). The countries represent a range of welfare‐state regimes and family systems in Europe, but because of the urban location of the focus groups, the research does not necessarily reflect rural discourses, which may be more conservative. Each focus group included 8–10 participants, with a total of 588 participants. The focus groups followed a standardized guideline (see Perelli‐Harris et al. 2014) to ensure that all groups discussed the same topics. The researchers transcribed the results in the native language of their countries, coded the results according to a standard procedure, and produced a country report in English that covered general topics. The collaborators then wrote an overview paper synthesizing the main findings of the project (Perelli‐Harris et al. 2014), as well as articles on each country. For the analysis below, we use the overview paper and the country reports, but we also asked country collaborators to revisit the transcripts and report on themes relating to the role of divorce in changing patterns of marriage and cohabitation.

### Quantitative data

To assess the relationship between the rise in divorce and cohabitation with quantitative data, we evaluate official statistics, survey data, and changes in divorce legislation. The official statistics were compiled in the Divorce Atlas and show the total divorce rate (TDR) (Spijker 2012). The TDR is “the mean number of divorces per marriage in a given year, or the divorce rate of a hypothetical generation subjected at each marriage duration to current marriage conditions” and can show period response to changes in policy. The individual survey data are based on female retrospective union histories from 16 surveys standardized in a dataset called the Harmonized Histories (Perelli‐Harris et al. 2010; and see www.ggp-i.org). Because men's histories were not available in all surveys, we show only women's experiences. The data come from nationally representative Generations and Gender Surveys (GGS) for the following countries and survey years: Belgium (2008–10), Bulgaria (2004), Czech Republic (2004–6), Estonia (2004–5), France (2005), Hungary (2004–5), Italy (2003), Lithuania (2006), Norway (2007–8), Poland (2010–11), Romania (2005), Russia (2004), and Sweden (2012–13). Because the GGS is not available for all countries (or the retrospective histories were not adequate for our purposes), we also used other data sources: the Dutch 2003 Fertility and Family Survey, the British Household Panel Survey (1991–2008), and the 2006 Spanish Survey of Fertility and Values. The surveys that comprise the Harmonized Histories have been frequently used in other studies and are generally considered high quality. In particular, fertility and marriage trends from most of the Generations and Gender Surveys reflect trends found in vital registration statistics (e.g., Vergauwen, Wood, and Neels 2015). Some countries such as the Netherlands interviewed only respondents of reproductive age, limiting the historical period we could analyze. Although we considered using earlier Fertility and Family Surveys for some countries, the age range surveyed (15–49) and the relatively small numbers of individuals at older ages did not allow for additional analyses.

### Divorce legislation reform

In order for divorce to be a precondition for the increase in cohabitation, divorce must first be legal. Changes in the legal availability of divorce, as well as the simplification of divorce requirements and procedures, contributed to the deinstitutionalization of marriage by allowing more couples to dissolve a marriage and by signaling the acceptability of marital dissolution (Lewis 2002; Cherlin 2004). The Appendix provides an overview of the dates of important divorce reforms in selected European countries.^1^ Most countries allowed spouses to divorce before 1950, and a majority of countries had also introduced divorce procedures that did not require fault to be established. Italy and Spain were the last countries to introduce or reintroduce divorce legislation in 1970 and 1981 respectively (divorce had been legal for some years during the Second Spanish Republic). Divorce by mutual consent was generally introduced only after fault‐based and no‐fault divorce procedures had become firmly established. In countries such as Austria, Czech Republic, and Sweden, decades or even centuries passed between the adoption of fault‐based divorce legislation and the introduction of divorce by mutual consent. Italy and Spain, in contrast, introduced divorce by mutual consent in conjunction with or a few years after fault‐based divorce procedures were introduced. Unilateral divorce by one spouse against the will of the other was introduced in some countries. In addition to the main divorce law reforms shown in the Appendix, many countries adopted further reforms that changed court procedures, waiting periods, or other requirements for divorce.

Previous research has shown that the implementation of no‐fault divorce led to a short‐term increase in crude divorce rates in European countries (González and Viitanen 2009; Kneip and Bauer 2009), possibly reflecting pent‐up demand due to changing attitudes toward marriage and gender roles. Pent‐up demand for divorce may also have led to divorce law reform; governments in some countries were increasingly pressured to enact divorce reform because so many couples had already separated. Hence, rates of union dissolution may have increased even without divorce reform. In any case, following the implementation of new divorce laws, legal divorce rates increased across most of Europe and the United States, although they may have leveled off more recently (Spijker 2012).

Couples can also separate without officially divorcing, and some of the earliest increases in cohabitation could have occurred because individuals were unable to divorce legally and wanted to live with a new partner (Burgoyne 1991; Kiernan and Estaugh 1993; McRae 1993). This earlier period of post‐separation cohabitation, however, was not widespread; we argue that only when divorce became legal and socially acceptable did marital dissolution foster the increase in cohabitation. Nonetheless, the distinction between legal divorce and separation is elided in many datasets; for example, survey questions ask respondents whether their parents lived together in childhood rather than asking specifically about marital status. The distinction between separation and divorce can also be blurred, since divorce is usually a process that includes separation and can last for years. Thus, below we primarily refer to divorce, but imply the general process of marital dissolution.

## Macro‐level links: The increase in divorce and the diffusion of cohabitation

Theories of the family often claim that the increases in divorce and cohabitation are part of the same set of family behaviors, emerging in conjunction with economic and social change. Women's increasing labor force participation and men's eroded position in the labor force have changed the marital bargain. As spouses come to resemble each other and gains from specialization are reduced, the value of marriage has deteriorated, with divorce becoming a more advantageous option for some (e.g. Becker 1991). Women's increased autonomy has also allowed women to postpone marriage and choose cohabitation as an alternative (Oppenheimer 1997; Kalmijn et al. 1997; Kalmijn 2011). The economic uncertainty and inequality that increased throughout the last decades of the twentieth century due to globalization (Blossfeld et al. 2006; Piketty 2014) exacerbated these trends: in many countries, individual‐level economic uncertainty is associated with union instability (Amato and James 2010) and with cohabitation and childbearing within cohabitation, particularly among the least educated (Perelli‐Harris et al. 2010; Hiekel et al. 2014). More broadly, social and ideational liberalization (Giddens 1992; Lesthaeghe 2010) led to greater emphasis on individualization and personal fulfillment and reduced the influence of institutions such as religion (Lesthaeghe and Surkyn 1988). Finally, the contraceptive revolution may have facilitated these developments by separating sex from reproduction, liberalizing sexual norms, and supporting feminism (Westoff and Ryder 1977). Hence, we recognize that many ideational and economic factors that are exogenous to the link between divorce and cohabitation may have independently led to the rise of both behaviors.

Nonetheless, the increase in divorce itself could have been one of the social changes that facilitated the increase in cohabitation. To explain the link at the macro level, we draw on diffusion theory, which has frequently been used to explain how the spread of new attitudes and behaviors led to the decline in fertility (Casterline 2001). Diffusion theory posits that changes in behavior occur through behavioral innovation, ideational change, and social dynamics. We argue that the increase in divorce is a relatively new social phenomenon that changes attitudes and spreads throughout social networks. The unprecedented and widespread prevalence of divorce altered social norms about the permanence of marriage, leading to its deinstitutionalization (Cherlin 2004; Lewis 2002). The relaxation of divorce laws with the implementation of no‐fault and mutual‐consent divorce reinforced the idea that divorce is acceptable and marriages can end. Marriage is then no longer perceived as an automatic way of organizing family life, but instead becomes more tenuous and uncertain. Cohabitation becomes an acceptable alternative living arrangement, particularly as a means of testing the relationship to ensure it is strong enough for marriage (McRae 1993; Perelli‐Harris et al. 2014; Hiekel and Keizer 2015).

### Explanations and mechanisms from qualitative research

Qualitative research has helped to elucidate this argument by providing further insights into possible mechanisms. Previous in‐depth interview research in the US found that individuals refer to a “fear of divorce” that leads them to be wary of the institution of marriage or to have doubts about marrying a particular individual (Miller et al. 2011). Qualitative evidence from the early 1990s in the UK also suggested that cohabitation emerged as a testing ground in response to divorce (McRae 1993). In our European focus group research, participants in nearly every country seemed to be aware of the link between divorce and cohabitation, stating for example that the rise in divorce and partnership instability was one of the main reasons for the increase in cohabitation (for full version with quotes, see Perelli‐Harris et al. 2016). In the Netherlands, this theme was so pervasive that Hiekel and Keizer (2015) argued that cohabitation was a strategic response to high marital instability. Participants from the UK focus groups also articulated an awareness that high divorce rates may discourage marriage. For some participants, the disillusionment with marriage led to a rejection of marriage altogether. In most countries, a few participants saw marriage as little more than a piece of paper.

At the same time, however, participants in most countries felt that marriage was still a sign of a committed relationship. As discussed in Perelli‐Harris et al. (2014), in all of the countries examined with the exception of eastern Germany, marriage was seen as valuable. The high value placed on marriage results in people wanting to test their relationship with cohabitation to ensure it is solid enough for marriage. Cohabitation was generally perceived as easier to dissolve than marriage, although in some cases children and mortgages could make a cohabiting partnership difficult to disentangle.

Focus group participants discussed several costs of divorce: psychological, emotional, social, financial, and bureaucratic. However, they agreed that divorce rarely incurred the same social stigma as it did in the past. Instead, participants were more likely to point out the financial, legal, or bureaucratic costs of divorce, such as the “fuss” involved in changing names and legal documents. The magnitude of the costs seemed to depend on the legal setting. In Italy, for example, participants in several focus groups mentioned the economic fear of divorce as well as the extensive court trials and long waiting periods. Overall, our analysis has shown that the general awareness and wariness of divorce has permeated throughout society and is a key factor leading to an increase in cohabitation at the macro level. Divorce has eroded some people's faith in marriage, leading them to eschew marriage altogether. At the same time, however, most participants still valued marriage and wanted to avoid the high costs and consequences of divorce. Thus, cohabitation plays an important role as a testing ground before marriage as a way of avoiding divorce.

### Analyses with quantitative data

Establishing the divorce/cohabitation link at the macro level is difficult given the potential over‐interpretation of observed correlations due to exogenous factors, such as female employment or ideational change. Nonetheless, examining basic trends is useful for showing how the two behaviors developed and seeing whether these qualitative discourses might be reflected in macro‐level data. When evaluating the quantitative data, we consider several criteria supportive of causal inference, but we primarily focus on temporal ordering (Ní Bhrolcháin and Dyson 2007). At a minimum, if increasing divorce rates lead to increasing levels of cohabitation, the rise in marital dissolution must occur first.

To evaluate the evidence that the increase in divorce preceded the increase in cohabitation, we compare three different indicators in Figure 1. Two of the indicators measure the increase in divorce, while one represents the general level of cohabitation in each country. The dark solid line represents the total divorce rate (TDR) and captures period “shocks” in divorce, for example due to changes in divorce law or economic conditions that may have curtailed divorce. In most countries, the TDR steadily increased throughout the period of observation, but it also reflects strong responses to divorce reform and socioeconomic change, for example in Russia, Estonia, Lithuania, and Spain.

Figure 1Total divorce rate, percent of ever‐divorced women among ever‐married women aged 30–49, and percent of currently cohabiting women among all couples aged 20–49 in four groups of countriesNOTE: See text for definition of total divorce rate.SOURCE: Harmonized Histories; Divorce Atlas.

**Figure:**
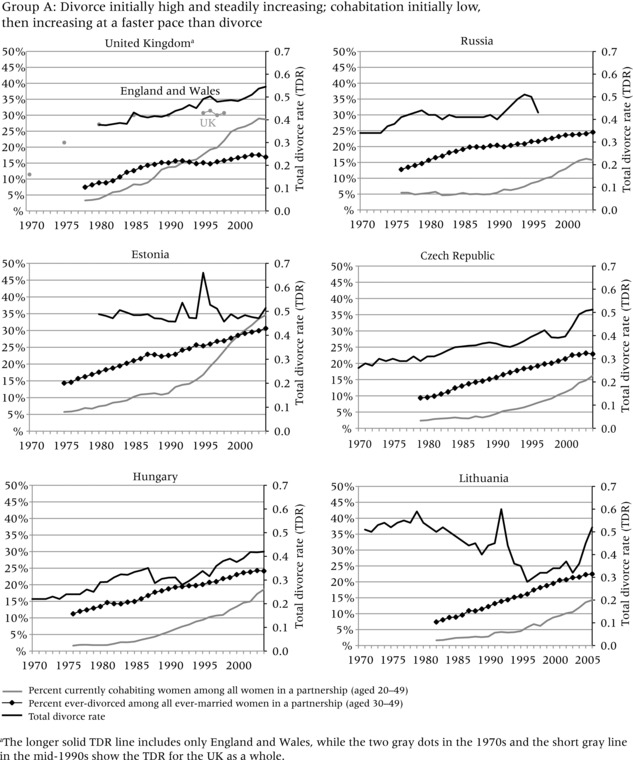

**Figure:**
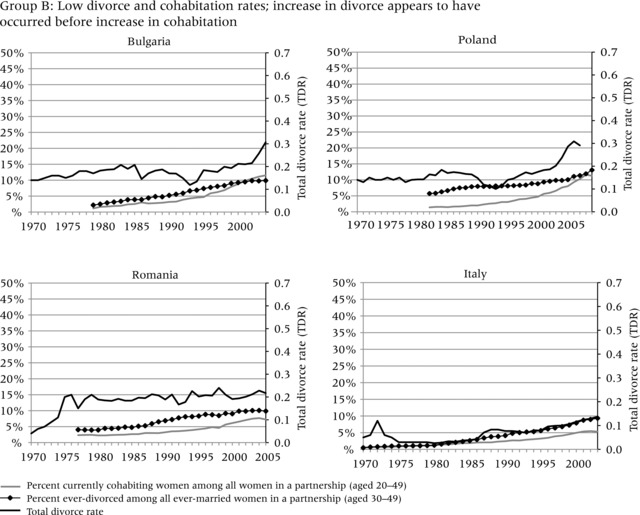

**Figure:**
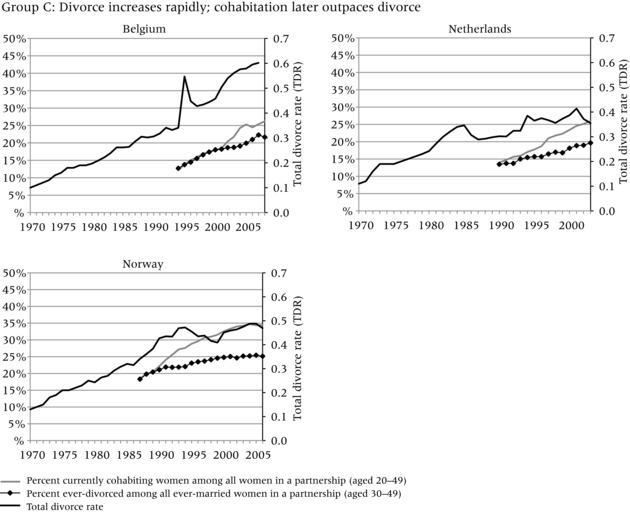

**Figure:**
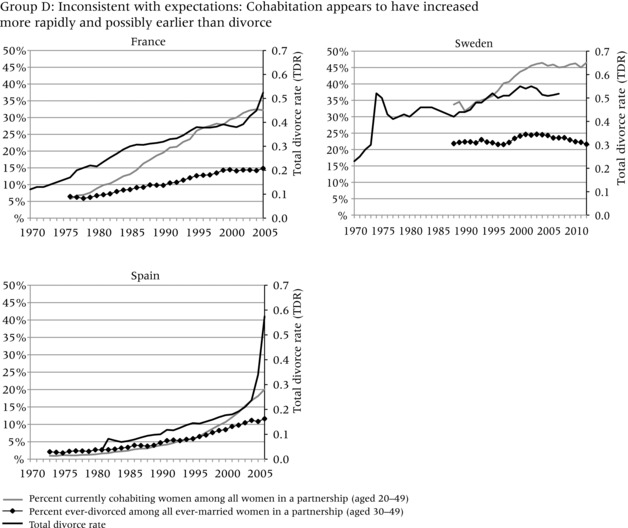

The diamond line, based on retrospective partnership histories, shows the percent of women who have ever experienced marital dissolution among all ever‐married women aged 30–49. This line represents the stock of those who ever divorced, or the share of the ever divorced in the general population. The gray line, also based on retrospective partnership histories, shows the percent of women aged 20–49 in a cohabiting relationship among those in partnership in a given year.^2^ This indicator shows how common cohabitation is during any given period.^3^ The trend lines start and end in different years in different countries, because survey years varied and each survey interviewed different age ranges and may not have interviewed sufficient numbers of older women to allow meaningful estimates for earlier years. To ensure sufficient numbers of women, each line starts in the year in which each age group includes at least 50 women. We only show women up to age 50; although we would have liked to include women who divorced later in life, the age 50 cutoff allows us to look farther back in time. Even with this age constraint, some countries still had only relatively short trend lines (e.g. in Belgium the trend line only starts in 1994, because insufficient women at older ages were interviewed).

In all countries, the increase in divorce preceded or coincided with the increase in cohabitation. The increase in the TDR always occurred before the increase in the percent currently cohabiting among those in a partnership, that is, it rose above 0.2 before the percent currently in cohabitation among those in partnership reached 10 percent (although the two increases seem to have occurred simultaneously in Spain). Nonetheless, the indicator for the percent ever divorced does not always have a straightforward relationship to the percent currently cohabiting. In addition, the magnitude of the change and the relative timing of the trends are more evident in some countries than others. To assist in interpretation of these different patterns, we cluster the countries into four groups. Group A, which has experienced a large increase in the prevalence of divorce and cohabitation, provides the strongest evidence that divorce facilitated the increase in cohabitation. Group B is characterized by relatively low divorce and cohabitation, but the increase in divorce still appears to have occurred before the increase in cohabitation. Group C shows very steep increases in the TDR and high levels of divorce and cohabitation, but data limitations in earlier periods make it difficult to conclude that divorce preceded cohabitation. Group D, finally, shows a pattern that seems to be inconsistent with the hypothesis: divorce increased substantially throughout or at the end of the observation period, but the increase in cohabitation appears to have occurred before the increase in divorce and may have developed for other reasons. We now discuss country trends in each of the four groups.

Group A contains countries in which divorce rates were already quite high by the 1970s and steadily increased thereafter. Cohabitation was initially low but increased at a faster pace than divorce. For example, the graph for the United Kingdom shows an increase in the total divorce rate^4^ over time, and the gray dots for the UK in 1970 and 1975, the only data available for that decade, suggest that divorce increased rapidly in the 1970s. This increase coincided with the reform of divorce laws in England and Wales (1971) and Scotland (1977), which allowed spouses to divorce by mutual consent and entitled one spouse to divorce unilaterally after five years of separation. The diamond line, based on partnership histories from the British Household Panel Survey, shows a pattern similar to that of the TDR, with the percent of ever‐married women who ever experienced divorce already increasing in the late 1970s. The percent of partnered women living in cohabitation started to increase only in the early 1980s (gray line), supporting the argument that the rise in divorce preceded the increase in cohabitation. Nonetheless, the percent of ever‐divorced women leveled off in the late 1980s, while the percent cohabiting continued to increase throughout the 1990s. The stabilization of divorce coupled with the increase in cohabitation may indicate that marriage is becoming more selective of stable relationships that are less likely to end in divorce (Beaujouan and Ní Bhrolcháin 2014; Berrington and Diamond 1999).

The post‐Socialist countries in Group A—Russia, Czech Republic, Hungary, Estonia, and Lithuania—also represent situations in which divorce was initially high and steadily increased, and cohabitation was initially low and then increased at a faster pace than divorce. In these countries, the total divorce rate was already relatively high—above 0.2—before the beginning of the observation period. In Russia, the TDR was above 0.3 in 1970, while in Lithuania it was closer to 0.5. In four of the five countries, the TDR stayed relatively high or increased, whereas in Lithuania the TDR decreased substantially, albeit with some short‐term peaks throughout the 1990s, possibly due to period shocks such as changes in policy. A similar peak in Estonia in 1995 coincided with a reform in divorce law, which allowed couples to jointly apply for divorce. The short‐term increase in the TDR in this year appears to reflect pent‐up demand for such an expedited divorce procedure. The legal change and the peak in the divorce rate may also have made people more wary of the institution of marriage, since we see a rapid increase in cohabitation rates after 1995. Thus, divorce and cohabitation increased in parallel during the 1970s and 1980s, but from the late 1990s cohabitation accelerated in all five countries. On balance, the evidence is consistent with the expectation that the increase in divorce facilitated the increase in cohabitation.

Group B contains three countries in Eastern Europe (Bulgaria, Poland, and Romania) and Italy. The figure shows cases in which divorce was initially low, then slowly increased, while cohabitation increased later in the 1990s and 2000s. These countries have maintained traditional family values and tend to be more religious (Reher 1998; Vignoli and Salvini 2014; Mynarska et al. 2014). Divorce was legalized much later in Italy, and mutual‐consent divorce is not available in Poland. Only recently have family behaviors started to change (Lesthaeghe 2010). In all four countries, the TDR hardly increased above 0.2, except for a slight increase to about 0.3 in the most recent years in Bulgaria and Poland. Cohabitation also remained low; the proportion of partnered women aged 20–49 who were cohabiting remained below 5 percent until the 2000s when it increased. However, even though these countries experienced only a moderate increase in the two behaviors until recently, divorce does seem to have increased before the rise in cohabitation, in accordance with our expectations.

The countries in Group C—Belgium, Netherlands, and Norway—represent cases in which divorce potentially increased before cohabitation, but it is difficult to reach definitive conclusions given the lack of data for earlier time periods. In all three countries, the TDR was below 0.2 in 1970 and sharply increased to above 0.4 by the late 1990s and early 2000s, indicating that these countries currently have high levels of divorce. Belgium's short‐term peak in the TDR in 1994 can be attributed to a reform that shortened divorce procedures. A further reform in 2000 reduced the necessary period of separation before divorce from five years to two. This may have contributed to the further increase in the TDR in the following years and the parallel increase in cohabitation. Note that the introduction of unilateral divorce procedures in Norway in 1993 did not have a similar effect. Instead, the TDR decreased briefly in the late 1990s. Here, however, de facto unilateral divorce had been previously granted by courts, resulting in little change in practice (Sverdrup 2002).

In the 1990s and 2000s, cohabitation increased rapidly in all three countries, with the rate of acceleration more rapid than for divorce. By the mid‐2000s the proportion of partnered women aged 20–49 who were cohabiting was around one‐quarter in Belgium and the Netherlands, and closer to one‐third in Norway. While the rate of increase in cohabitation was faster than the rate of increase in divorce in the 1990s or 2000s, the lack of comparable data on cohabitation during the 1970s and 1980s means we can only speculate that divorce increased earlier than cohabitation. Earlier surveys from Norway suggest that cohabitation was relatively rare in the 1970s but increased rapidly throughout the 1980s, while divorce was well established by the 1970s (Noack 2001). Prior estimates reflect similar developments in the Netherlands; divorce started to increase in the late 1960s and accelerated in the 1970s, while cohabitation became more widespread in the 1980s (Latten 2005). In Belgium, census and register estimates suggest that the percent cohabiting was very low throughout the 1980s and only started to increase in the 1990s (Corijn 2005). Divorce rates, on the other hand, started to increase in the 1970s, well before cohabitation became acceptable (Matthijs 1988). Thus, earlier studies in these countries support our claim that divorce increased before cohabitation.

Group D represents three countries—France, Sweden, and Spain—whose experience does not appear to be consistent with the claim of temporal ordering, although all three have experienced steep increases in both divorce and cohabitation. In France, the TDR steadily increased over the three decades, from below 0.2 to above 0.5, but the percent of currently cohabiting couples rapidly outpaced the percent ever divorced, suggesting that cohabitation may have developed independently. Sweden's TDR rose sharply in 1975 following a major divorce reform that entitled spouses to demand divorce without a reason, even against the will of the partner after a maximum waiting period of six months. The TDR remained relatively high in the years after this reform; however, the Swedish survey data show that the percent currently cohabiting was much higher than the percent ever divorced at the beginning of the observation period and continued to increase much more rapidly than divorce. Data from the 1992 Swedish Family Survey show that cohabitation was already widespread in 1975; 71 percent of women aged 20–24 in unions were cohabiting, but this proportion declined to 15 percent for 30–34‐year‐olds and eventually most people did marry (Bernhardt 1998). Because divorce rates were relatively high in Sweden, especially after the 1974 reforms, it is likely that the two behaviors started to increase in parallel. Finally, Spain is difficult to categorize. Divorce only became available in 1981, and levels of cohabitation and divorce remained low during most of the period. Both increased in the early 2000s, and the TDR increased sharply in 2005 following a legal reform that allowed divorce without a period of previous separation. However, because the increase in cohabitation appears to have accelerated in 1995, we are reluctant to say that divorce definitely increased before cohabitation.

Thus, the evidence for a macro‐level link between divorce and cohabitation is stronger in some countries than others. In some countries cohabitation increased earlier than divorce, as in France and Sweden, and more recently in Spain. We do not have focus group data for these countries to see whether the explanations for the increase in cohabitation were substantially different from those in other countries. Overall, however, our results suggest that in most countries the initial increase in divorce preceded the increase in cohabitation.

## Meso‐level links: The intergenerational transmission of parents’ divorce/children's cohabitation

Studies in many countries have found that parental divorce is a strong predictor of children's divorce (e.g. Amato 1996; McLanahan and Sandefur 1994; Wagner and Weiß 2006; Dronkers and Härkönen 2008; Wolfinger 2005) and children's cohabitation (e.g., Axinn and Thornton 1992, 1996; Amato 1996; Bumpass, Sweet, and Cherlin 1991; Thornton 1991; Berrington and Diamond 2000; Liefbroer and Elzinga 2012; Wolfinger 2005). Parents’ marital dissolution can change children's attitudes and decisions about relationships through a process of social learning (Cui and Fincham 2010; Smock et al. 2013) and socialization (Axinn and Thornton 1996). The experience of parental divorce may lead children to be more accepting of alternatives to life‐long marriage, reduce the perceived rewards of marriage, and make children more reluctant to enter committed relationships (McRae 1993; Amato 1996; Axinn and Thornton 1992, 1996; Cui and Fincham 2010; Dronkers and Härkönen 2008). Research in the US has also shown that parents’ experience of cohabitation, especially after divorce, is positively associated with adult children's own cohabitation, since they would have observed their parents choose this arrangement (Sassler et al. 2009; Smock et al. 2013).

### Explanations and mechanisms from qualitative research

The intergenerational transmission of parents’ divorce/children's cohabitation emerged repeatedly in the focus groups. Individuals whose parents divorced stated that they were unlikely to marry and would choose cohabitation instead. Both the previous sociological literature on the intergenerational transmission of family behaviors and the focus group discussions point to cohabitation as a way to cope with parental marital breakdown and the ensuing skepticism about marriage. Focus group participants were aware that parental separation often leads individuals to reject the institution of marriage, or at the very least to cohabit first to see whether their relationship will last. The qualitative research shows how attitudes, and indeed strong emotions, are an important mechanism in understanding the divorce/cohabitation link.

### Analyses with quantitative data

**Figure 2:**
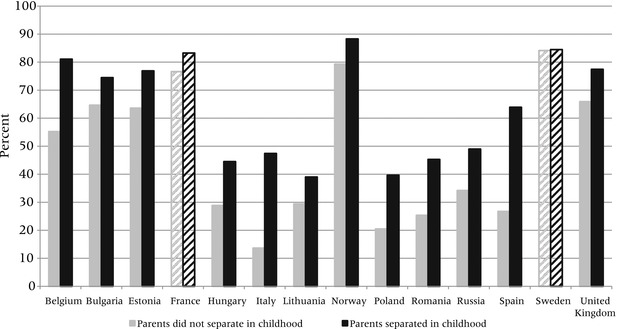
Percent of ever‐partnered women aged 20–49 in 2005 who started their first union with cohabitation (compared to direct marriage), by parents’ union status at women's age 15 NOTES: Solid bars indicate significant differences (non‐overlapping confidence intervals) between parents' union at age 15 for those whose first union type is cohabitation. Diagonal bars indicate no significant difference. Weights applied if available. Years of analysis may differ depending on survey. SOURCE: Harmonized Histories.

To examine the divorce/cohabitation link at the meso level, we use the Harmonized Histories to ascertain whether people whose parents separated or divorced are more likely to enter cohabitation (rather than direct marriage) for their first partnership compared to people whose parents remained married in childhood. This analysis allows us to directly investigate the causal link based on temporal ordering: by definition, parental divorce when children are young occurs before the latter make decisions about their first union. Figure 2 shows the proportion of ever‐partnered women aged 20–49 in 2005 who started their first union with cohabitation rather than marriage by whether their parents lived together when the women were aged 15. This measure was obtained from a survey question that is relatively consistent across countries.^5^

In all countries except Sweden, the proportion of women who began their first union with cohabitation was higher for those whose parents separated than for those whose parents did not. The difference between the two groups is significant in all countries except France and Sweden. In most countries, about 10 percent more women started their unions with cohabitation among those whose parents separated compared to those whose parents did not separate. The figure also indicates that direct marriage (marriage without prior cohabitation) has remained more common among those whose parents stay married. In Sweden, France, and Norway, however, fewer than 25 percent of couples directly married, indicating that cohabitation is now the normative way of entering a co‐residential partnership.^6^

Because we look at women aged 20–49 in 2005, these analyses reflect cohorts who were aged 15 in 1971–2000. In some countries, divorce legislation and the increase in divorce would have occurred earlier and would not be reflected here. In addition, selectivity into cohabitation may have declined over time as cohabitation became more normative. As a check, we repeated the analyses for each ten‐year age group and found the same relationship; hence, the relationship is not due to the increase in both cohabitation and experience of parental separation across cohorts. Ideally we would have liked to repeat our analyses for the same age group in earlier years when divorce had just started to emerge in some countries; however, given the small sample sizes in most countries, this was not possible. Nonetheless, these results are consistent overall with the idea that the intergenerational transmission of parents’ divorce/children's cohabitation is common across countries, and that intergenerational transmission can be considered a potential causal pathway helping to explain the link between divorce and cohabitation.

## Micro‐level links: Individual experience of divorce and subsequent cohabitation

At the micro level, an individual's own experience of divorce may lead to a preference for cohabitation for subsequent unions. People who had a negative experience with their first marriage may be more likely to live together without marrying than those who were still influenced by the traditional norms of their families. This may be the case especially in countries with high divorce rates, but also in countries where marriage occurs at a young age, for example in countries east of Hajnal's line (Coale 1992). In Hungary, Spéder (2005) found that post‐divorce cohabitation drove the spread of cohabitation, with premarital cohabitation only emerging since the 1980s. Researchers in other countries have also speculated that the rise in cohabitation began with the previously married, for example in France (Villeneuve‐Gokalp 1991) and the UK (Haskey 1994; Kiernan and Estaugh 1993; Burgoyne 1991).

### Explanations and mechanisms from qualitative research

In all countries, focus group participants noted how their own experience of divorce led to skepticism about marriage and a preference for cohabitation in second unions. People who had divorced recounted their difficult experiences in the court systems, the expense, and bureaucratic frustrations. Hence, personal experience of divorce often produces a dislike of the institution of marriage and the choice of cohabitation for second unions and raises the question of whether cohabitation may have emerged first among those who experienced divorce.

### Analyses with quantitative data

Cross‐national quantitative research shows that second unions in many European countries are more likely to start with cohabitation than with marriage, even in countries with a low prevalence of cohabitation (Galezewska 2016). These findings provide evidence that cohabitation is the preferred type of union for those who have previously been married. Here, however, we are interested in whether the majority of cohabiting couples have previously divorced relative to those who were never married.^7^ By examining the percent of currently cohabiting women who were previously divorced, we can ascertain the extent to which divorced individuals were the forerunners of the cohabitation boom. In line with our temporal ordering hypothesis, we expect that the divorced would make up a substantial share of cohabitors at the beginning of the observation period, and this share would stay the same or decline over time as the never‐married group became more prevalent.

Table 1 shows the percent of previously married women (i.e. divorced) aged 20–49 among all those who are currently cohabiting in a given five‐year period for all countries except Belgium. In any given period, a relatively small proportion of cohabitors were previously married; most were never married. In nearly every country, over 75 percent of those cohabiting were never married. For the most part, contrary to our hypothesis, cohabitation seems to have increased more among the never married than among the divorced. Nonetheless, in Hungary, Russia, and Estonia over one‐third of cohabiting women experienced divorce in some of the periods of observation. As discussed above, many former Socialist countries had a relatively young age at first marriage and high levels of divorce, resulting in a larger group exposed to repartnering. These divorced women may have discarded strong marriage traditions and cohabited rather than married. Hence, post‐marital cohabitation may have played a substantial role in the increase in cohabitation in these countries.

**Table 1:** Percent of currently cohabiting women aged 20–49 who were previously married by 5‐year time period

	1978–82	1983–87	1988–92	1993–97	1998–02	2003–07
Bulgaria	6.0	11.4	13.8	14.8	14.0	13.8
Czech Republic	18.6	19.8	22.2	25.3	22.2	19.8
Estonia	35.7	36.2	34.5	29.4	23.7	21.2
France	14.8	14.2	12.5	11.6	9.3	9.5
Hungary	29.2	38.2	36.1	32.8	24.7	19.0
Italy	15.4	20.2	15.3	14.4	14.1	14.3
Lithuania	‐	15.8	19.2	18.1	19.9	20.0
Netherlands	‐	‐	12.5	11.5	10.0	10.4
Norway	‐	12.5	14.7	14.6	14.3	13.0
Poland	‐	15.7	23.0	20.5	16.7	16.9
Romania	9.9	16.9	15.4	15.7	15.6	15.5
Russia	30.6	34.5	34.0	36.1	34.3	34.3
Spain	5.8	12.9	18.0	16.1	13.0	10.9
Sweden	‐	‐	10.7	8.7	8.8	8.3
United Kingdom	25.1	16.5	12.4	14.1	11.9	11.6

NOTE: Belgium not shown, as the time series only starts in 1994. All data weighted, apart from that for Czech Republic, Poland, and Sweden where weights are not available. Results are based on 5‐year information centered on January of each year. Final data range (2003–2007) centered around 2005 or the latest year for which data are available.

SOURCE: Harmonized Histories.

Other countries may also have experienced a higher proportion of previously married women cohabiting when cohabitation was just starting to become widespread. The UK, for example, had a greater proportion of cohabitors who had divorced when cohabitation was just starting to increase than it had in later periods (Table 1). In 1978–82, 25 percent of cohabiting women had previously divorced, suggesting that the initial increase in cohabitation may have been led by the newly divorced. Subsequently, the relative proportion who had divorced declined, with those who had never married representing a much greater proportion of those currently cohabiting. This decline seems to have occurred in other countries as well, or, more commonly, the proportion of the divorced relative to the never married remained relatively stable at around 15 percent. Partially this may be due to the age range analyzed; our analyses capture women only up to age 49, and cohabitation among the previously married may have initially increased more at older ages. Note also that we do not have early estimates for Norway, Sweden, and the Netherlands, when cohabitation was practiced by less than 15 percent of the population. In general, however, with the exception of the UK, Hungary, Russia, and Estonia, these results suggest that the divorced were not the primary forerunners of cohabitation, although they may have played a small role in the increase as cohabitation began to diffuse.

## Discussion

Throughout the industrialized world, divorce and cohabitation rapidly emerged from the 1970s onward, buoyed by social, economic, and ideational change. Although exogenous factors such as rising female employment and ideational change no doubt played a key role in spurring these trends, divorce and cohabitation also seemed to be increasing through interactions with each other—in particular, divorce may have been a catalyst for the increase in cohabitation. We explored explanations and evidence for this link at the macro, meso, and micro levels. Our quantitative results were consistent with the argument that divorce played an important role in facilitating cohabitation at all three levels, but the evidence was stronger at some levels than others. The meso‐level evidence was most consistent, indicating strong intergenerational transmission of divorce in all but two countries (Sweden and France). The macro‐level evidence was partially consistent, with support for the causal link in 13 of the 16 countries, but little support in Sweden, France, and Spain. The micro‐level results, however, did not fully support the claim that the previously divorced drove the increase in cohabitation, although prior research suggests that the previously married have a greater likelihood of cohabiting than the never married (Galezewska 2016). We now reflect on this evidence and provide an argument for why the rise in divorce may be linked to the increase in cohabitation.

On the macro level, the diffusion of attitudes and norms about divorce and marriage may have been one of the key causal pathways leading to the increase in cohabitation. The focus group research suggests that Europeans are cognizant of the consequences of divorce and that the possibility or experience of divorce may discourage people from marrying, or at least from marrying quickly and without ensuring that the relationship is solid. These discourses arose in all of our study countries, but were especially prevalent in the Netherlands and the UK. Participants mentioned that cohabitation is a way to test the relationship in order to avoid the costs of divorce, which were usually described as higher than the costs of cohabitation dissolution. In fact, some participants—especially in Italy, Germany, and Austria where waiting times are long and divorce procedures more difficult—complained about the high costs of divorce, saying that they refused to marry as a result. For these participants, cohabitation was seen as a favorable alternative to marriage. Nonetheless, except in eastern Germany, most focus group participants did not eschew marriage altogether and planned to marry in the future; for them, cohabitation was seen as a way to make sure their relationship was strong enough to get married (Perelli‐Harris et al. 2014).

Our quantitative macro analyses partially supported this diffusion argument. In most of the observed countries, the increase in divorce occurred before the increase in cohabitation, indicating the appropriate temporal ordering for causality, although direct causality was impossible to determine. In the remaining countries, the two behaviors increased simultaneously. In some countries, the increase in divorce rates was preceded by legal reforms that introduced (in Spain and Italy) or simplified divorce procedures. In some countries such as Belgium and Sweden, legal reforms had an immediate and clearly discernible effect on divorce behavior. In others, legal reforms did not have a direct impact on the TDR, but may have contributed to changing attitudes toward marriage and cohabitation in the longer term. The magnitude of the increase and the strength of the link between divorce and cohabitation also differed among countries. In some countries, such as the UK, Russia, Estonia, Lithuania, and Hungary, divorce was widespread before cohabitation, suggesting that high levels of divorce may have changed social norms and attitudes about the institution of marriage. In France and Sweden, on the other hand, cohabitation emerged early on, and the increase outpaced divorce so rapidly that most likely other important factors led to the diffusion of cohabitation. In countries such as Belgium, Netherlands, and Norway, divorce increased rapidly from the 1970s, but we cannot conclude definitively that the increase in divorce preceded the increase in cohabitation. Nonetheless, in most of our countries, divorce seems to be a necessary but insufficient cause for the diffusion of cohabitation, and the explanations for the increase in cohabitation may be context specific.

On the meso level, substantial evidence suggests that the link between divorce and cohabitation may have been driven by the intergenerational transmission of attitudes and behavior. Parental divorce emerged repeatedly in the focus group discussions, with the children of divorced couples often blunt in their rejection of marriage or at least direct marriage. This finding was reflected in the quantitative analyses: respondents whose parents separated during their childhood were significantly more likely to choose cohabitation for their first union than those whose parents remained married, with the exception of France and Sweden, where nearly everyone enters cohabitation rather than directly marrying. These results suggest that on the meso level, the diffusion of cohabitation occurred through the process of social learning: as individuals observed the dissolution of their parents’ marriage, they may have adopted behaviors and attitudes that led them to be more skeptical of marriage and committed relationships in general (Cui and Fincham 2010; Axinn and Thornton 1996). These individuals may then have preferred cohabitation as an alternative living arrangement, allowing them to live with a partner without committing to a more permanent relationship. Our findings suggest that social learning from parents’ experience was one of the most important pathways for the diffusion of cohabitation.

We also found evidence on the micro level indicating that divorced individuals may prefer to cohabit when repartnering, because of their negative personal experience with marriage. The tendency to choose cohabitation after divorce was a widespread theme in our focus groups. Most divorced individuals held a very low opinion of marriage and expressed a reluctance to remarry. Previous quantitative research supports these assertions by showing that throughout Europe, second and higher‐order unions are much more likely to start with cohabitation than direct marriage and that the propensity to cohabit is greater among the previously married than the never married (Galezewska 2016). Overall, these findings indicate that the institutional constraints of marriage and high costs of divorce dissuade people from marrying, but not necessarily from repartnering through cohabitation. Nonetheless, the quantitative analyses showed that the previously married did not appear to be the forerunners of cohabitation in most countries. In three countries with relatively high divorce rates—Russia, Hungary, and Estonia—over one‐third of cohabiting women were divorced in some periods, but the majority of cohabitors were never married. Even so, while the overall increase in cohabitation may have been driven by the never married rather than the divorced, the role of cohabitation for those who did divorce could still have been very important: individual experiences of divorce may have influenced others’ perspectives on marriage and revealed the advantage of cohabitation before marriage.

While this project is a first step in understanding whether and how divorce and cohabitation are related, it has several limitations. The qualitative research had broad coverage by being conducted in eight countries in Europe, but it was not conducted in all of our study countries for which we also have quantitative data and clearly does not fully reflect all European diversity, especially in rural areas. It would be particularly interesting to see whether divorce is an important theme in Sweden, Spain, and France or whether other explanations for the increase in cohabitation predominate instead. Also, several of our hypotheses are implicitly about the initial rise in cohabitation, and we cannot go back far enough in time to explore norms and attitudes when cohabitation was just beginning to emerge. This is also the case for some countries for the quantitative analyses: to our knowledge, data on cohabitation in the 1970s and 1980s are lacking for several of our countries, so we cannot know for sure which emerged first—divorce or cohabitation. Finally, our analyses lack covariates or controls for selection effects, which may be critical for revealing confounders or explaining differences across countries. Our goal was to provide a broad, descriptive overview, but more sophisticated analyses may provide robust evidence for or against a causal link.

This study provides fundamental insights into how divorce has altered social norms and facilitated the increase in cohabitation to the extent that it has become an alternative way of living with a partner. Previous conceptualizations of family change have often assumed that family behaviors change in tandem. Our study, however, has demonstrated that the increase in one behavior can potentially lead to the emergence of another. Indeed, the timing and sequencing of the rise in divorce and cohabitation raises questions about whether all aspects of family change are linked to the same underlying phenomena, and to what extent these phenomena are the same across countries. Nonetheless, while other exogenous factors may have been important for the increase in cohabitation, our study shows that the rise in divorce, with its concomitant shifting of attitudes and perceptions about marriage, may well have been a crucial catalyst for the increase in cohabitation.

## Supporting information

Supporting Information.

## Notes

1 Appendix is available at the supporting information tab at wileyonlinelibrary.com/journal/pdr.

2 The percentages were estimated in January of a given year. Weights have been applied if available.

3 The increase in divorce may have led to the delay or avoidance of co‐residential unions, but we are less interested in the decision to remain single than in whether people chose cohabitation over marriage.

4 The longer solid TDR line includes only England and Wales, while the two gray dots in the 1970s and the short gray line in the mid‐1990s show the TDR for the UK as a whole.

5 In the UK, the question referred to age 16. The question was not asked in the Netherlands.

6 Note that these results do not control for potential covariates. Our data do not provide further detail about the parents’ partnerships, including the experience of cohabitation or the formation of step‐families.

7 Also relative to those widowed. The widowed make up a relatively small share across countries, and the share remains stable over time.
