# Supplementary material for: The Rise in Divorce and Cohabitation: Is There a Link?
Source: Popul Dev Rev. 2017 Jun 5;43(2):303–29. doi: 10.1111/padr.12063 (PMC5518209; doi:10.1111/padr.12063)
Supplement: Supplementary file 1 — Supporting Information. [file PADR-43-303-s001.docx]

**Appendix**

We distinguish between dates when divorce first became available; no-fault divorce procedures simplified divorce; procedures became available for mutual-consent divorce; and divorce by unilateral decision was introduced. These different types of divorce are not mutually exclusive and have been available within the same jurisdictions at different points in time.

TABLE A1 Important divorce law reforms in 16 European countries

|  | **Legalization of divorce** | **Introduction of no-fault divorce** | **Introduction of divorce by mutual consent** | **Introduction of unilateral divorce** |
| --- | --- | --- | --- | --- |
| Belgium | before 1950 | before 1950 | before 1950 | 1975 |
| Bulgaria | before 1950 | before 1950 | until 1952 and since 1968 |  |
| Czech Republic | before 1950 | before 1950 | 1998 |  |
| Estonia | before 1950 | before 1950 | 1969 | 2010 |
| France | before 1950 | 1976 | 1976 | before 1950 and 1976 |
| Hungary | before 1950 | before 1950 | until 1953 and since 1974 |  |
| Italy | 1970 | 1975 | 1975 | 1970 |
| Lithuania | before 1950 | before 1950 | 1970 |  |
| Netherlands | before 1950 | 1971 | 1971 |  |
| Norway | before 1950 | before 1950 | before 1950 | 1993 |
| Poland | before 1950 | before 1950 |  |  |
| Romania | before 1950 | before 1950 | 1993 | 2010 |
| Russia | before 1950 | before 1950 | 1965 | before 1950 and 1996 |
| Spain | 1981 | 1981 | 1981 | 1981 |
| Sweden | before 1950 | before 1950 | before 1950 | 1974 |
| United Kingdom |  |  |  |  |
| England and Wales | before 1950 | 1971 | 1971 | 1971 |
| Scotland | before 1950 | 1977 | 1977 | 1977 |
